# Expression profile of long non-coding RNA in inner Mongolian cashmere goat with putative roles in hair follicles development

**DOI:** 10.3389/fvets.2022.995604

**Published:** 2022-09-02

**Authors:** Rong Ma, Fangzheng Shang, Youjun Rong, Jianfeng Pan, Min Wang, Shuran Niu, Yunpeng Qi, Yanbo Li, Qi Lv, Zhiying Wang, Ruijun Wang, Rui Su, Zhihong Liu, Yanhong Zhao, Zhixin Wang, Jinquan Li, Yanjun Zhang

**Affiliations:** ^1^College of Animal Science, Inner Mongolia Agricultural University, Hohhot, China; ^2^Key Laboratory of Mutton Sheep Genetics and Breeding, Ministry of Agriculture and Rural Affairs, Hohhot, China; ^3^Key Laboratory of Animal Genetics, Breeding and Reproduction, Inner Mongolia Autonomous Region, Hohhot, China; ^4^Engineering Research Center for Goat Genetics and Breeding, Inner Mongolia Autonomous Region, Hohhot, China

**Keywords:** cashmere goat, hair follicle, lncRNA, expression profile, functional analysis

## Abstract

The hair follicle is a complex skin accessory organ, which determines hair growth. Long non-coding RNAs (lncRNAs) have been proven to play an important role in hair follicle development, but their specific mechanism is still unclear. In this study, high-throughput sequencing was used to obtain the expression profiles of lncRNA in the hair follicles of Inner Mongolian cashmere goats at different embryonic stages (45, 55, 65, and 75 days), and a total of 6,630 lncRNA were identified. According to the rules of hair follicle development, we combined miRNA and mRNA databases (published) and predicted lncRNA-miRNA, miRNA-mRNA, and lncRNA-mRNA interaction pairs in the 45 vs. 75 comparison group. We obtained 516 lncRNA-mRNA, 1,011 lncRNA-miRNA, and 7,411 miRNA-mRNA relationship pairs. Finally, target genes were analyzed by Gene Ontology (GO) and Kyoto Encyclopedia of Genes and Genomes (KEGG), and it was found that they were mainly enriched in the Wnt signaling pathway and PI3K-Akt signaling pathway related to hair follicle development, indicating that lncRNA may interact with miRNA/mRNA to directly or indirectly regulate the expression of genes related to hair follicle development. Dual-luciferase reporter gene analysis showed that *lncRNA MSTRG.1705.1* could bind to *Chi-miR-1*, while *lncRNA MSTRG.11809.1* had no binding site for *Chi-miR-433*. In conclusion, this study aims to further analyze the molecular regulation mechanism of hair follicle development and to lay a theoretical foundation for revealing the regulation mechanism of cashmere hair follicle growth.

## Introduction

Inner Mongolia cashmere goat is a distinctive local breed in China. Cashmere has a white texture and good luster and is the main raw material for the textile industry. Because cashmere fabrics are warm, smooth, comfortable, and beautiful (1), they are loved by the public and sold all over the world. The hair follicle is a complex skin accessory organ, which determines the production and quality of cashmere (2). It can be divided into primary hair follicles that grow coarse hairs and secondary hair follicles that grow cashmere. The development of hair follicle morphology involves a series of interactions between epithelial cells and dermal fibroblasts, and the process is complex (3, 4). First, the mesenchymal cells send signals to induce the epidermis to form hair germ, and then hair germ releases some factors inducing the formation of the dermal fibroblast and the dermal papilla; finally, the dermal papilla releases a “second signal” to promote the proliferation and differentiation of epithelial cells, thereby forming hair follicles with complete structure (2–6). The results showed that the morphogenesis of both primary and secondary hair follicles in cashmere goats was initiated in the embryonic period, but the morphogenesis of primary hair follicles was earlier than that of secondary hair follicles (7). At the embryonic stage of 45 days, the skin has formed a complete epidermal structure, the hair follicle structure has not yet appeared, and the keratinocytes are arranged in the basal layer of the epidermis (8). And primary hair follicles begin to develop at the embryonic stage of 55 days, and mature at the embryonic stage of 135 days; secondary hair follicles begin to occur at the embryonic stage of 75 days, and they can mature from 6 months after birth (8, 9).

LncRNA is a class of RNA with more than 200 nucleotides that cannot encode proteins but have a poly-A tail structure similar to mRNA (10, 11). Initially, due to its lack of a complete open reading frame (ORF) and no protein-coding function, it was considered a by-product of RNA polymerase II transcription, without biological functions (12). However, there is evidence that lncRNA plays critical and diverse regulatory functions in a variety of physiological processes (13–18). For example, lncRNAs can regulate transcription, epigenetic modifications, protein/RNA stability, translation, and post-translational modifications by interacting with DNA and/or proteins (19–21). In addition, lncRNAs can also interact with RNAs to regulate gene expression. For example, it can bind to mRNAs to directly regulate mRNA expression and competitively bind the same miRNA with mRNA to indirectly regulate gene expression. For example, *lncRNA Pvt1* can inhibit the expression of C-Myc protein, thus inhibiting the development of autosomal dominant polycystic kidney disease (22); *LncRNA PINK1* competitively binds to *miR-203* with *ATF2*, thereby aggravating cerebral ischemia/reperfusion oxidative stress injury (23). lncRNA not only plays an important role in human diseases but also plays an indispensable role in the growth and development of hair follicles. For example, *lncRNA PLNCRNA-1* can promote the proliferation and differentiation of hair follicle stem cells and shorten the cell cycle (24); The expression level of *lncRNA H19* in the anagen phase of cashmere was significantly higher than that in the catagen and telogen phase of cashmere, indicating that *IncRNA H19* may play an important role in the development of cashmere (25); Overexpression of *lncRNA-000133* could significantly increase the relative expression levels of *ET-1, SCF, ALP*, and *LEF1* in dermal papilla cells (26). In this study, high-throughput sequencing was used to establish the expression profiles of lncRNA in the hair follicles of inner Mongolian cashmere goats at different embryonic stages (45, 55, 65, and 75 days), and 6 lncRNAs were randomly selected to verify the database. In addition, we constructed lncRNA-mRNA and lncRNA-miRNA-mRNA interaction networks by combining the existing miRNA and mRNA data, and the binding relationship between *lncRNA MSTRG.1705.1* and *Chi-miR-1* or *lncRNA MSTRG.11809.1* and *Chi-miR-433* were verified by dual-luciferase reporter analysis. Finally, target genes involved in regulatory networks were analyzed by the GO and KEGG. We found that they were mainly enriched in cell proliferation and cell differentiation pathways (GO), as well as in the Wnt signaling pathway and PI3K-Akt signaling pathway (KEGG). Studies have shown that the Wnt signaling pathway and PI3K-Akt signaling pathway were important signaling pathways involved in hair follicle morphological development. Therefore, we speculate that lncRNAs regulate the development of hair follicles by directly or indirectly regulating genes related to hair follicle development and then regulating the proliferation, apoptosis, and differentiation of hair follicle cells.

## Materials and methods

### The sample collection

In this experiment, 12 3-year-old ewes (cashmere-bearing goats in Aerbasi, Inner Mongolia) with good production performance and the same growth environment were selected for synchronous estrus treatment and their mating time was recorded (experimental animals were from Inner Mongolia Jinlai Animal Husbandry Technology Co., Ltd.). All animal experiments were carried out following the guidelines for Experimental Animals of the Ministry of Science and Technology (Beijing, China). According to the records of mating time, 12 fetal skin samples were collected at 4 periods (45, 55, 65, and 75 days), immediately treated with DPEC water, and placed in liquid nitrogen. The samples were stored in the −80°C freezer in the laboratory for later use. All samples were collected by the International Guiding Principles for Biomedical Research Involving Animals and were approved by the experimental animal ethics committee of Inner Mongolia Agricultural University.

### RNA library construction and sequencing

Total RNA was isolated and purified using Trizol reagent (Invitrogen, Carlsbad, CA, USA) according to the manufacturer's instructions. Subsequently, the total RNA concentration and quality were detected by NanoDrop ND-1000 and Agilent 2100, and 12 RNA libraries were constructed using qualified total RNA. Remove ribosomal RNA from total RNA according to instructions of the Ribo ZeroTM rRNA Removal Kit (Illumina, San Diego, California, USA). And the remaining RNA fragments were reverse transcribed to form the final cDNA using the RNA seq Library Prep Kit (Illumina). Finally, we performed the paired-end sequencing on an Illumina Hiseq 4000 (LC Bio, Hangzhou, Zhejiang, China) following the protocol recommended by the supplier.

### Quality control, comparison, and splicing of lncRNA

Cutadpter (27) software was used to control the quality of raw data, sequently removing reads containing adapters, ploy-N, and reads of low quality. FastQC was used to verify the sequence quality. Bowtie 2 (28) and Hisat 2 (29) were used to compare the valid data with the reference genome. Finally, the mapped reads of each sample were assembled by StringTie (30).

### Identification and differential analysis of lncRNA

According to the characteristics of lncRNAs, we identified lncRNA transcripts following the conditions: their length was ≥200 bp, the number of exons was ≥1, and their read coverage was >3. Subsequently, CPC (31) and CNCI (32) were used to predict transcripts with coding potential, all transcripts with CPC score ≤0.5 and CNCI score ≤0 were excluded, and the remaining transcripts were considered as lncRNAs. In addition, we classified potential lncRNAs into the following five categories: potentially novel isoform (j), a transfer falling entirely within a reference intron (i), generic exonic overlap with a reference transcript (o), intergenic transcript (u), exonic overlap with reference on the opposite strand (x). Finally, the FPKM value was used to estimate the expression level of lncRNA, and the effects of sequencing depth, gene length, and differences between samples on gene expression could be eliminated by FPKM treatment (33). In this experiment, R package-edge was used to screen differentially expressed lncRNAs under the conditions of |log2foldchange| ≧ 1 and *P*-value < 0.05.

### Validation of sequencing results by qRT-PCR

Total RNA was extracted from fetal skin samples of Inner Mongolia cashmere goats at four embryonic stages (45, 55, 65, and 75 days) using Trizol reagent (Takara, Dalian, Liaoning, China) according to the manufacturer's instructions. Subsequently, RNA was reverse transcribed to cDNA using the PrimeScript RT Reagent Kit with gDNA Eraser (Takara, Dalian, Liaoning, China). Finally, the qRT-PCR experiment was performed on the LightCycler^®^96 Real-Time PCR system (Roche, Basel, Switzerland), using TB GreenPremix Ex Taq II (Takara, Dalian, Liaoning, China), β-actin as an internal reference. The qRT-PCR conditions were: 95°C for 10 min, 95°C for 15 s, and then 40 cycles at 60°C for 30 s, followed by 72°C for 10 s. All experiments were performed in three duplicates, and the relative expression levels of genes were calculated using the 2^−ΔΔCt^ method (34).

### Construction of lncRNA-mRNA interaction networks

To better understand the functions of differentially expressed lncRNAs, we predicted the target genes of lncRNAs. Target genes can be divided into cis and trans according to the distance and expression correlation between lncRNAs and protein-coding genes. Due to the large results of trans-regulation and the difficulty of later verification, in this project, we mainly analyze the genes that are regulated in cis. When the expression correlation of lncRNA and its target gene is ≥0.95 and the distance is <100 kb, it is considered a potential target. Based on the relationship between differentially expressed lncRNAs and mRNAs, the lncRNA-mRNA regulatory network was constructed. Finally, Cytoscape (35) was used to visualize it.

### Construction of lncRNA-miRNA-mRNA interaction networks

In this study, Targetscan (http://www.targetscan.org/mamm_31/) and miRanda (http://www.microrna.org/microrna/home.do) software were used to predict lncRNA-targeted miRNAs and miRNA-targeted mRNAs, and constructed lncRNA-miRNA-mRNA regulatory networks. Finally, it is visualized using Cytoscape (35).

### Dual-luciferase reporter assays

*Chi-miR-433* mimics and *Chi-miR-1* mimics were synthesized by Hanbio Biotechnology Company (Shanghai, China). The wild/mutated fragment of *lncRNA MSTRG.11809.1* that can bind to *Chi-miR-433* and the wild/mutated fragment of *lncRNA MSTRG.1705.1* that can bind to *Chi-miR-1* were inserted into psiCHECK2 to generate psiCHECK2-lncRNA MSTRG. 11809.1-WT/MUT and psiCHECK2-lncRNA MSTRG.1705.1-WT/MUT vector. Subsequently, psiCHECK2-lncRNA MSTRG.11809.1-WT/MUT was co-transfected with *Chi-miR*-433 mimics, and psiCHECK2-lncRNA MSTRG.1705.1-WT/MUT was co-transfected with *Chi-miR-1* mimics. At 48 h post-transfection, luciferase activity was measured using a dual-luciferase reporter assay system.

### Enrichment analysis

Subsequently, in order to elucidate the potential roles of target genes in lncRNA-mRNA and lncRNA-miRNA-mRNA, GO and KEGG enrichment analysis of target genes was performed. The GO database is used to predict the function of gene products in molecular functions, biological processes, and cellular groups (36). The KEGG database can analyze the biochemical metabolic pathways and signal transduction pathways involved in genes (37). When the *P*-value of the GO item and KEGG pathway is < 0.05, it is considered to be significantly enriched.

### Statistical analysis

All data were expressed as mean ± SEM, and the results were evaluated using Student's *t*-test. A value of *P* < 0.05 was considered statistically significant.

## Results

### Evaluation of RNA-sequencing data

Total RNA was extracted from the fetal body skin of Inner Mongolia cashmere goat at 45, 55, 65, and 75 days, and the concentration and quality of the total RNA samples were detected. The total RNAs of the 12 samples were all within the range of 1.8 < OD260/OD280 <2.4, 1.5 < OD260/OD230 <2.4, and 7 ≤ Rin <10, indicating that the extracted total RNA was not degraded and was of good quality, which can be used for subsequent experiments.

In this study, high-throughput sequencing was performed at four stages of the fetal phase of Inner Mongolian cashmere goats (Albas type). we constructed 12 libraries that removed ribosomal RNA of cashmere goats during the fetal period, obtaining 1 063 299 566 original reads. Subsequently, 1 023 889 360 effective reads were obtained after quality control of the original reads. The mean value of quality control data in each period is shown in Table 1: The Q20 (the proportion of bases with quality value ≥20, error rate < 0.001) is between 99.97 and 99.98%, and the Q30 (the proportion of bases with quality value ≥30, error rate < 0.001) was between 98.33 and 98.49%. It shows that the probability of bases being misidentified during the sequencing process is very low and can be ignored, and the sequencing results are reliable.

**Table 1:** Data quality control statistics.

**Sample**	**Raw data**	**Valid data**	**Valid ratio (reads)**	**Q20%**	**Q30%**	**GC content %**
d45	84,983,821	81,905,808	96.43	99.97	98.36	45.83
d55	95,674,074	91,909,209	96.07	99.98	98.49	46.83
d65	91,375,373	8,811,8172	96.44	99.97	98.33	46.83
d75	82,399,920	79,363,264	96.31	99.98	98.41	45.33

### Sequence alignment

We used Hisat (29) to compare 1 023 889 360 valid read and reference genomes, and averaged the results for each period. the percentage of the number of reads on the reference genome as a percentage of valid reads was more than 96%, and the percentage of the number of reads compared with the unique location of the reference genome as a percentage of valid reads was more than 77%, and the number of reads compared with the multiple locations of the reference genome as a percentage of valid reads was more than 17% (Table 2). The results showed that the data utilization was normal, and the obtained raw data met the requirements of subsequent lncRNA analysis.

**Table 2:** Reference genome alignment read statistics.

**Sample**	**Valid reads**	**Mapped reads**	**Unique mapped reads**	**Multi mapped reads**
d45	81,905,808	78,683,181 (96.03%)	63,754,282 (77.83%)	14,928,899 (18.20%)
d55	91,909,209	89,600,112 (97.49%)	74,763,405 (79.01%)	16,988,907 (18.49%)
d65	88,118,172	85,760,995 (97.33%)	69,442,089 (78.73%)	16,318,906 (18.60%)
d75	79,363,264	77,350,953 (97.46%)	63,404,799 (79.89%)	13,946,154 (17.57%)

### Identification and characterization of lncRNA

**Figure 1:**
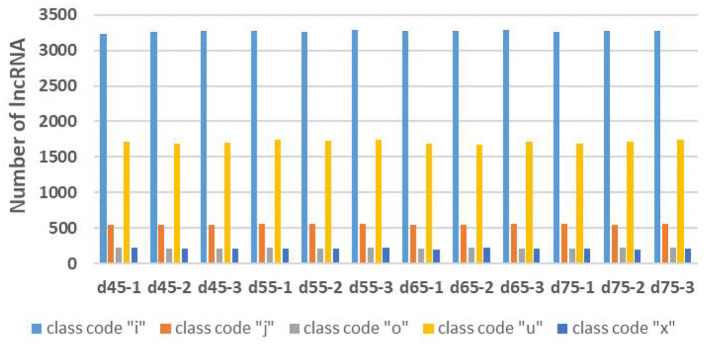
The statistics of different types of lncRNAs in the embryonic stage of Inner Mongolia cashmere goat. The blue column represents the “i” class lncRNA, the orange column represents the “j” class lncRNA, the gray column represents the “o” class lncRNA, the yellow column represents the “u” class lncRNA, and the dark blue represents the “x” class lncRNA.

**Figure 2:**
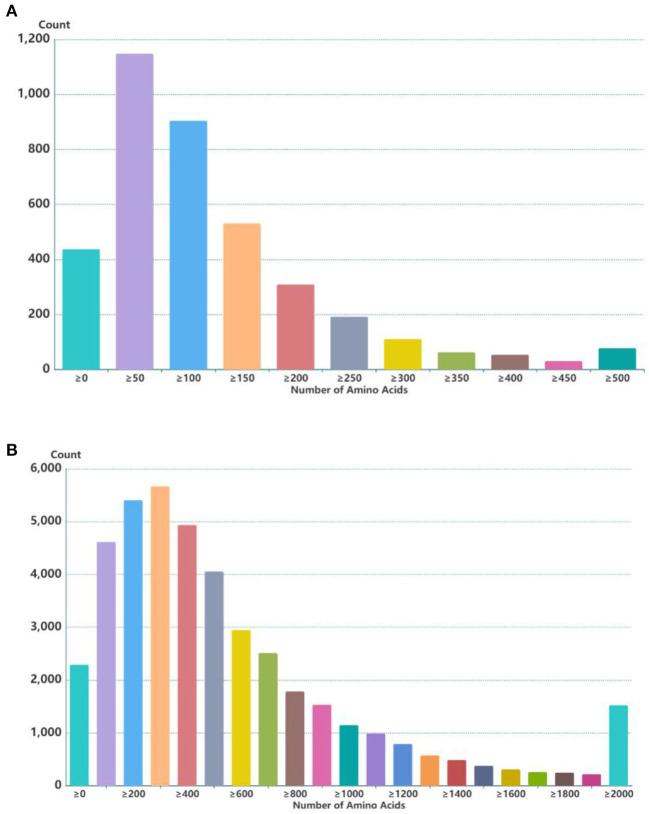
ORF length statistics of lncRNA and mRNA in embryonic hair follicles of Inner Mongolia cashmere goats. **(A)** ORF length of lncRNA. **(B)** ORF length of mRNA.

**Figure 3:**
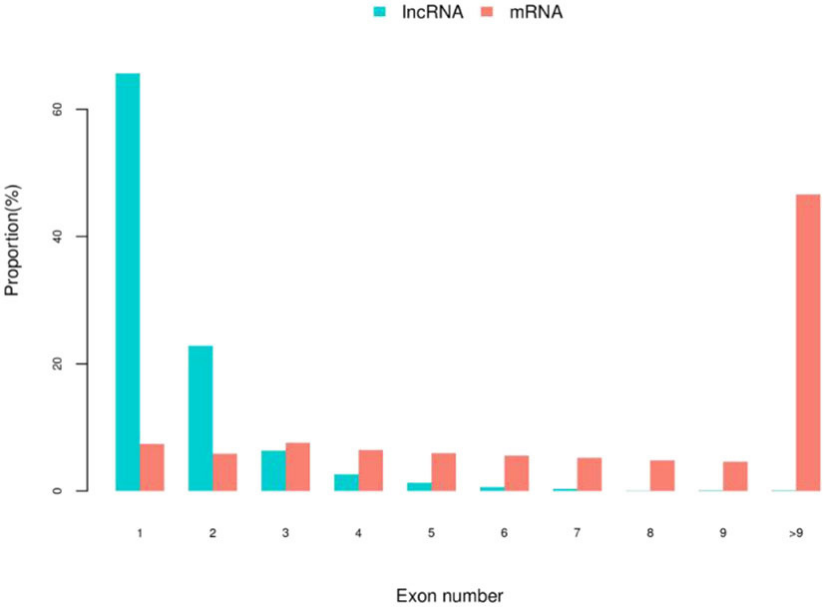
The statistics of lncRNA and mRNA exons number in embryonic hair follicles of Inner Mongolia cashmere goats. The blue column represents the number of lncRNA exons, and the red column represents the number of mRNA exons.

**Figure 4:**
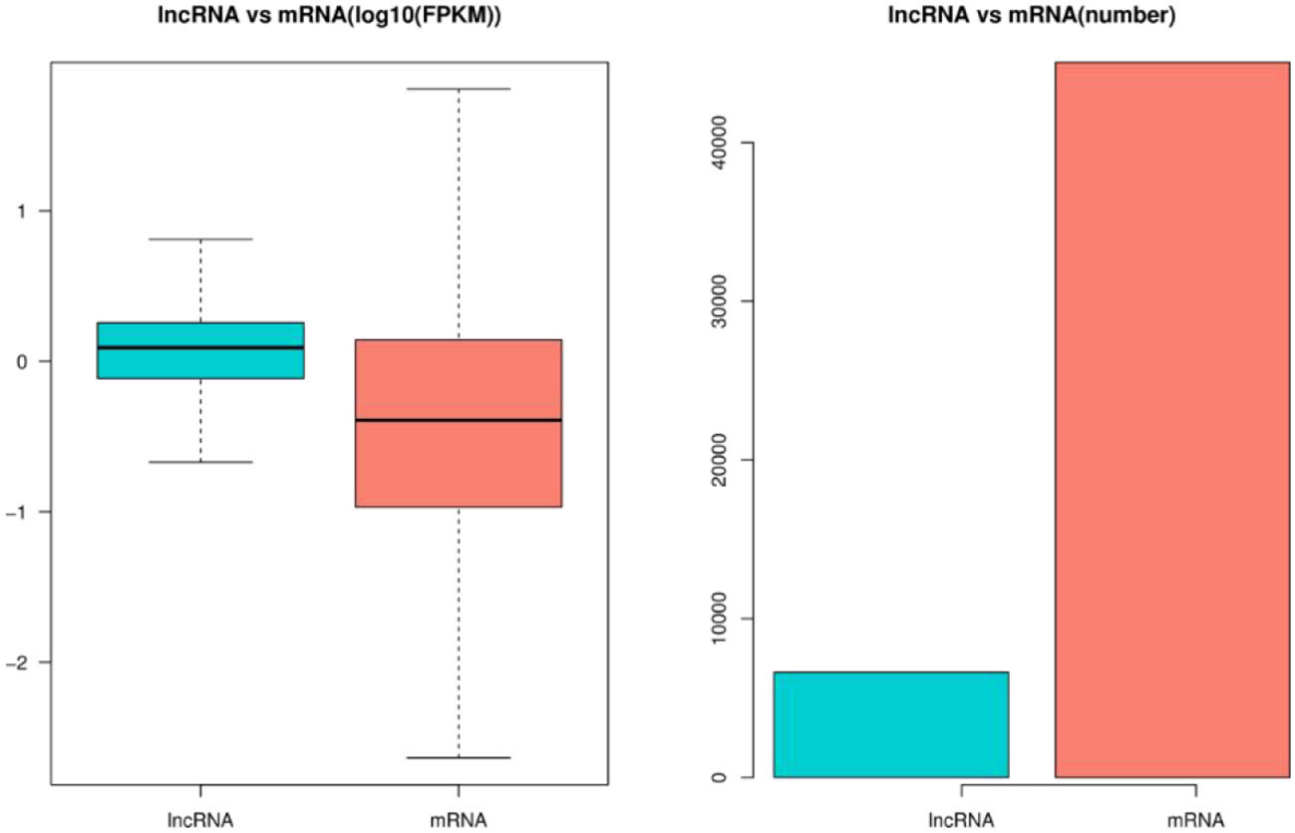
Expression levels of lncRNA and mRNA in embryonic hair follicles of Inner Mongolia cashmere goat. The blue column represents the average expression level of lncRNA, and the red column represents the average expression level of mRNA.

Through the screening and identification of transcripts, a total of 6,630 lncRNA transcripts were finally identified. By classifying lncRNAs, it was found that most of the obtained lncRNAs were “i” and “u” types, which accounted for 52.43% and 30.60% of all lncRNAs, respectively (Figure 1). From the perspective of structural characteristics (Figures 2, 3), both lncRNA and mRNA have ORF and exon, but the ORF of lncRNA is usually shorter (compared to mRNA), and the number of exons is far less than that of mRNA. Finally, the analysis of the number and expression level of lncRNA and mRNA (Figure 4) showed that the obtained lncRNA number and its expression level were far lower than the mRNA level (mRNA database published).

### Differential expression analysis of lncRNAs

**Figure 5:**
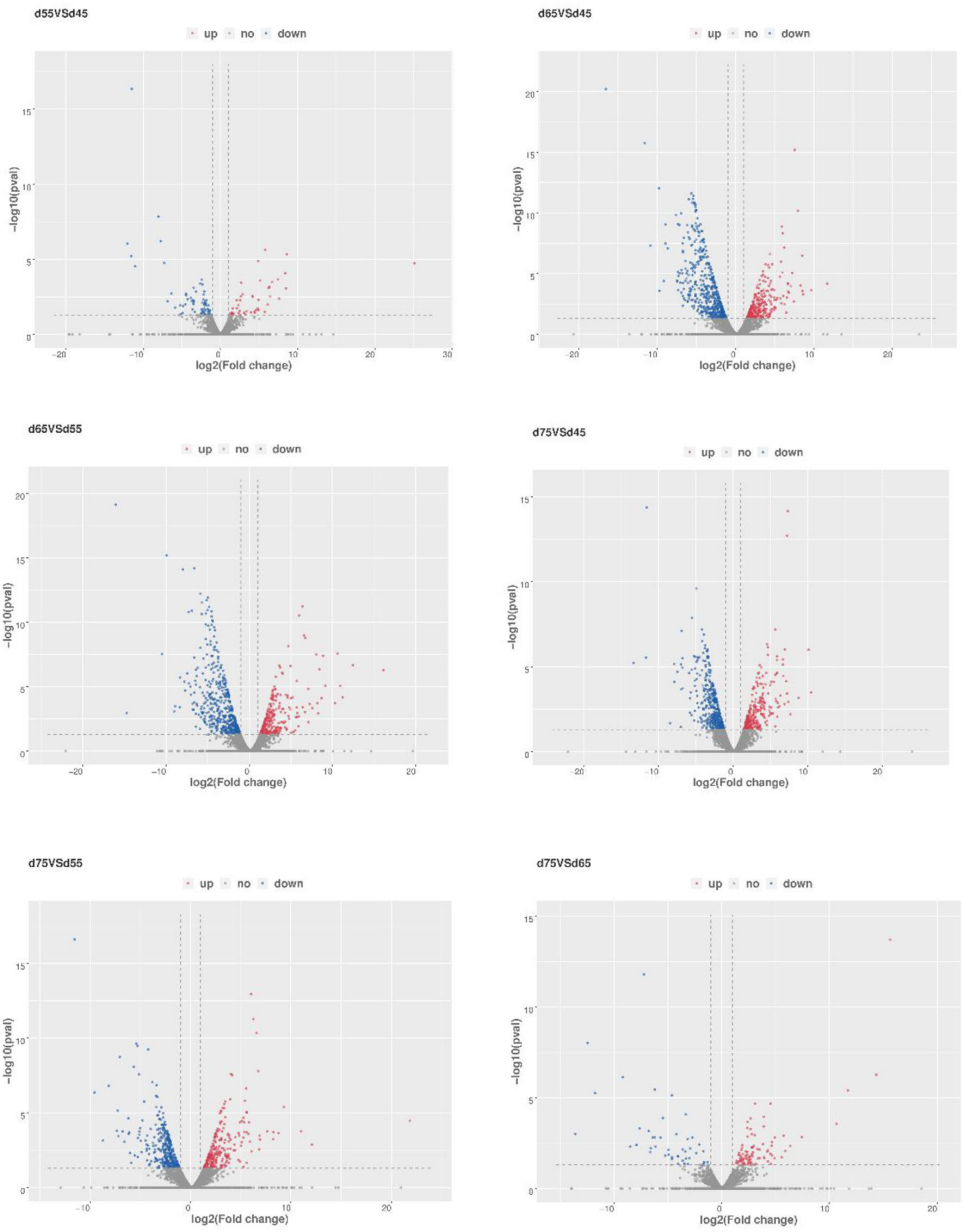
Transcriptional analysis of differentially expressed lncRNA in different embryonic stages of Inner Mongolia cashmere goats. The blue dots represent differentially expressed and downregulated lncRNAs, the red dots represent differentially expressed and upregulated lncRNAs and the gray dots represent lncRNAs with no significant difference in expression.

**Figure 6:**
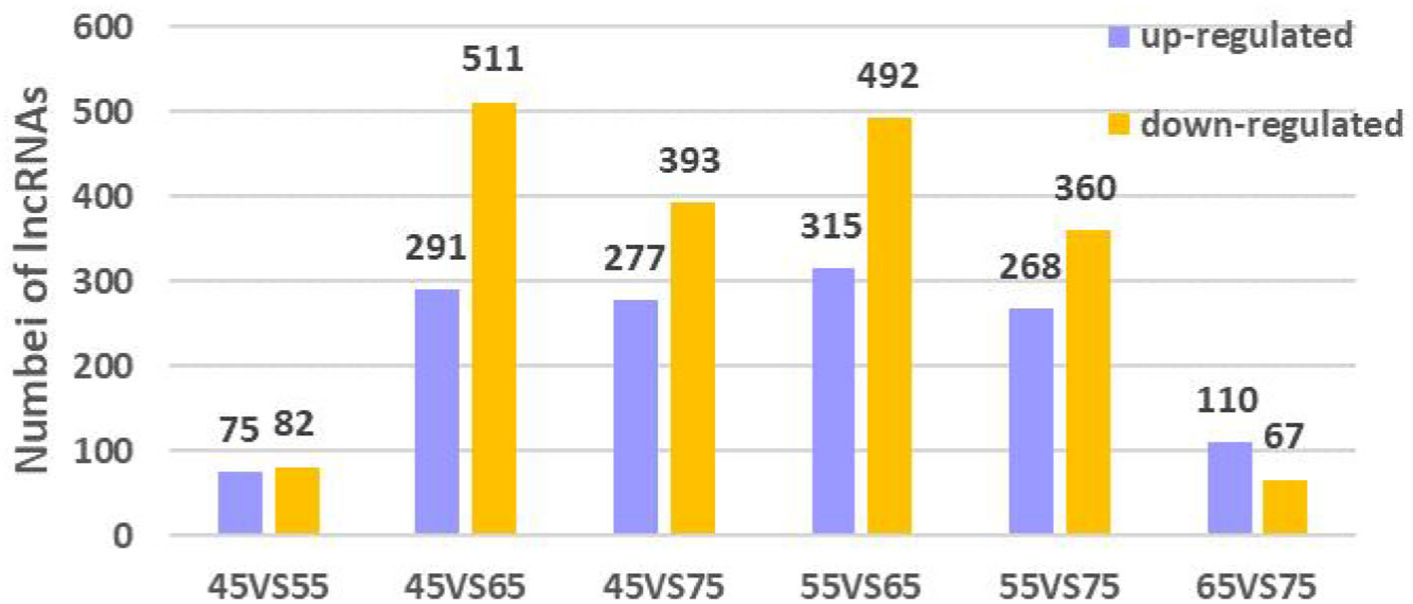
Differential lncRNA expression in different embryonic stages of Inner Mongolia cashmere goats. The purple columns represent upregulated lncRNAs, and the orange column represents downregulated lncRNAs.

To further explore the regulatory role of lncRNAs in the development of hair follicles, we divided the four stages into six comparison groups, with |log2foldchange| 1 and *P*-value < 0.05 as the conditions for screening differential lncRNAs (Figure 5). The results are as follows: d45 vs. d55, lncRNA upregulated by 75 and downregulated by 82; d45 vs. d65, lncRNA upregulated by 291 and downregulated by 511; d45 vs. d75, lncRNA upregulated by 277 and downregulated by 393; d55 vs. d65, lncRNA upregulated by 315 and downregulated by 492; d55 vs. d75, lncRNA upregulated by 268 and downregulated by 360; d65 vs. d75, lncRNA upregulated by 110 and downregulated by 67 (Figure 6).

### Validation of RNA sequencing using qRT-PCR

**Figure 7:**
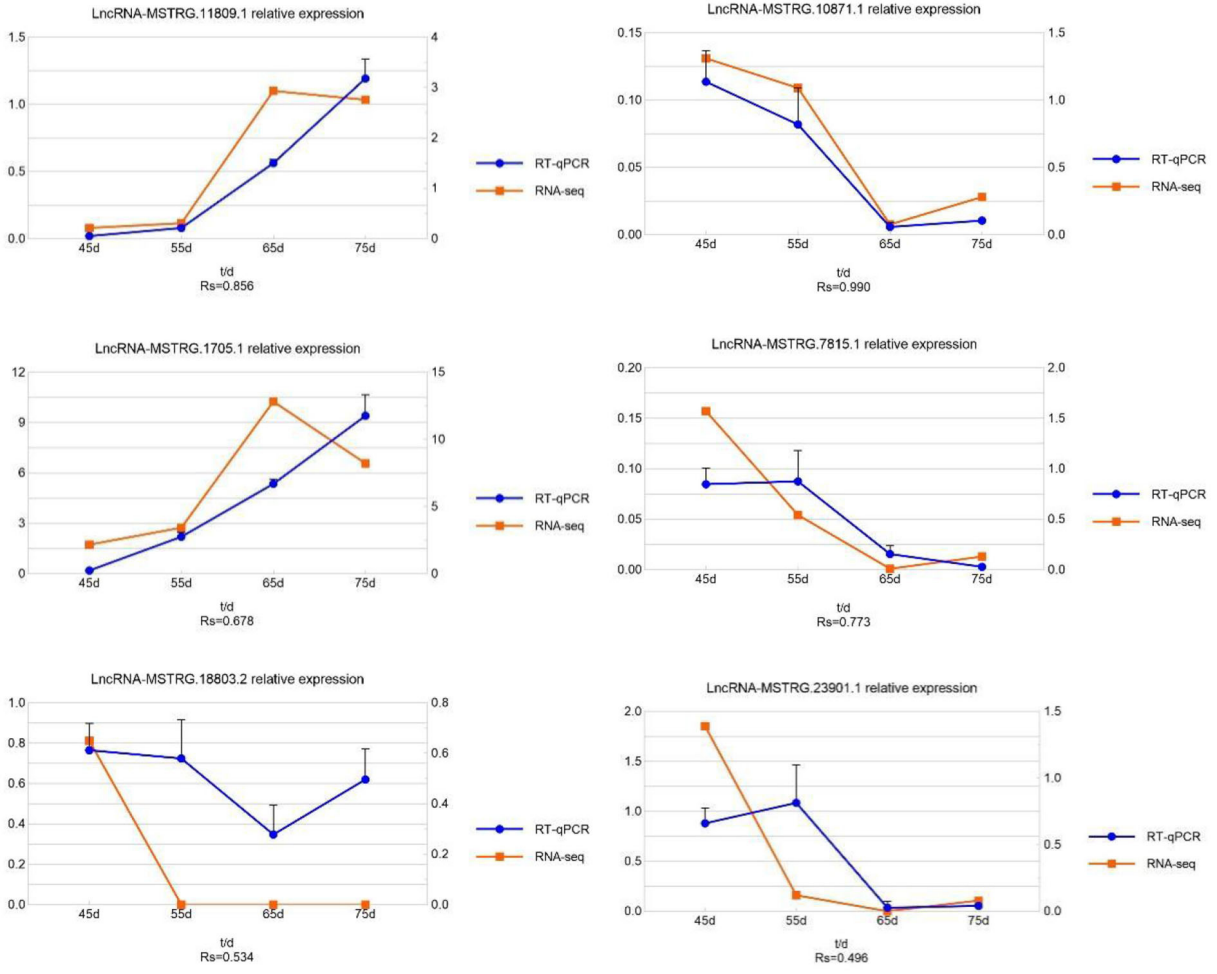
The expression quantity and expression trend of lncRNA in different periods. A total of 0.8 < | Rs| < 1 indicates a strong correlation; 0.6 < | Rs| < 0.8 indicates a strong correlation; 0.4 < | Rs| < 0.6 indicates a moderate correlation; 0.2 < | Rs| < 0.4 indicates a weak correlation; 0 < | Rs| < 0.2 indicates no correlation; and the degree of proximity between | Rs| and 1 represents the degree of closeness and correlation between two variables.

To verify the reliability of the lncRNA sequencing results, we randomly selected six lncRNAs (*lncRNA MSTRG.11809.1, lncRNA MSTRG.10871.1, lncRNA MSTRG.1705.1, lncRNA MSTRG.7815.1, lncRNA MSTRG.18803.2, lncRNA MSTRG.23901.1*) for qRT-PCR. The experimental results are shown in Figure 7. The qRT-PCR results were consistent with the sequencing results, indicating the sequencing results are accurate and reliable.

### LncRNA–mRNA network construction

**Figure 8:**
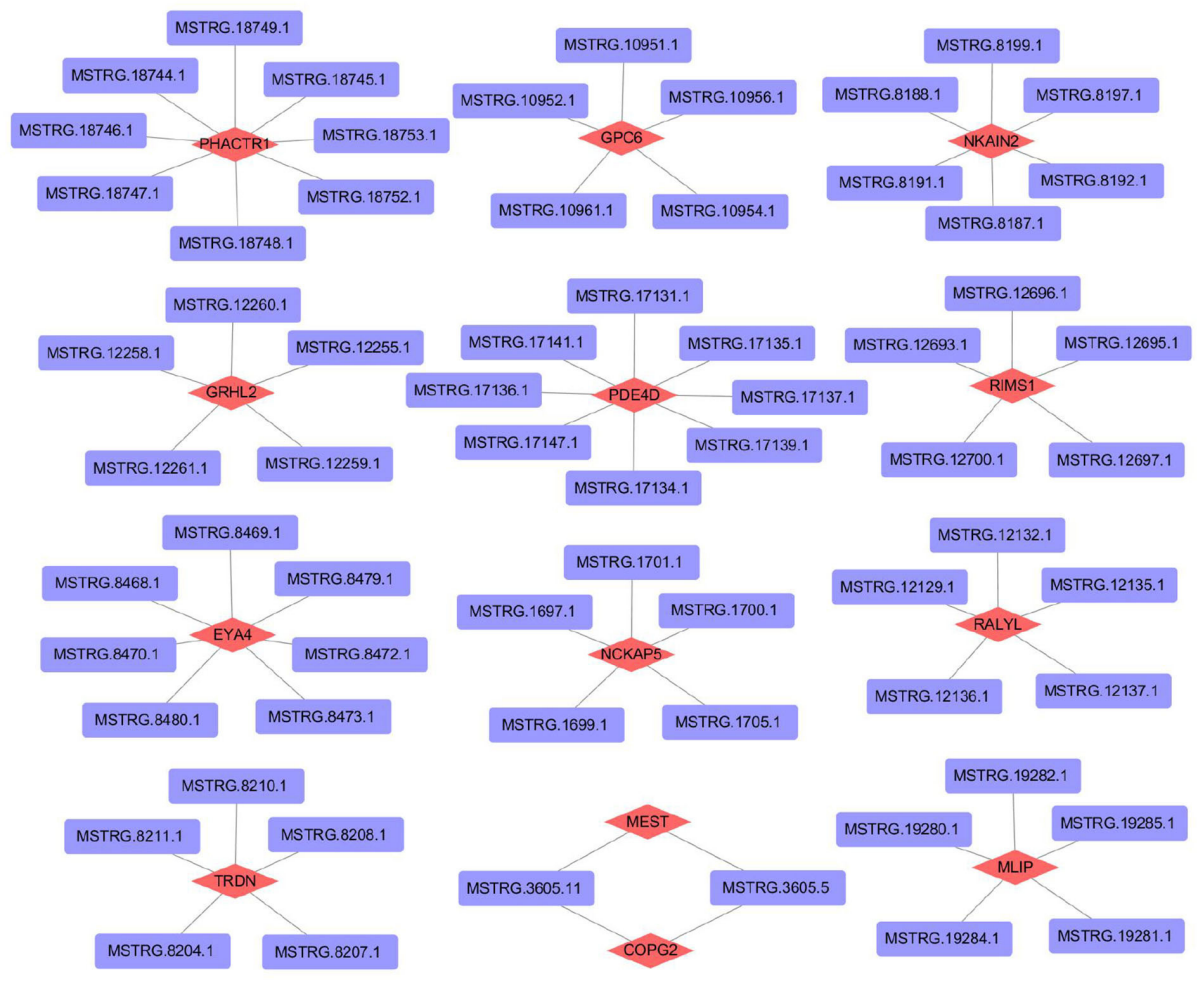
Construction of lncRNA -mRNA regulatory network related to hair follicle development in Inner Mongolia cashmere goats.

To further understand the regulatory mechanism of lncRNAs, we selected differentially expressed lncRNAs in the 45VS75 comparison groups to predict target genes (combined with existing mRNA data), and constructed lncRNA-mRNA co-expression networks (Supplementary Table 1). A total of 516 lncRNA-mRNA regulatory networks were predicted, of which 449 lncRNAs and 316 mRNAs were involved in the construction of regulatory networks. Partial prediction results are shown in Figure 8.

### Functional analysis of lncRNA as a miRNA sponge

**Figure 9:**
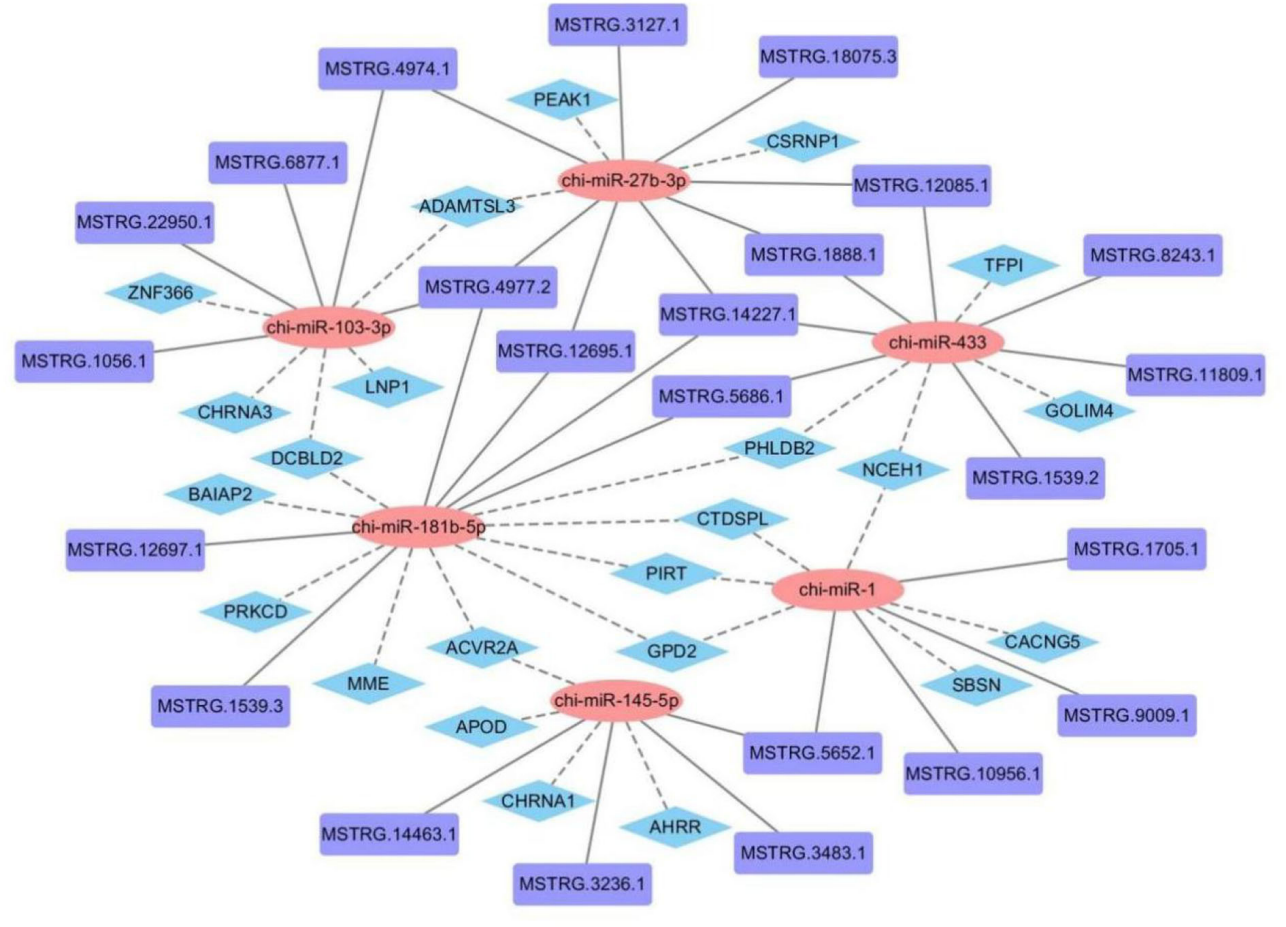
Construction of lncRNA-miRNA-mRNA regulatory network related to hair follicle development in Inner Mongolia cashmere goats.

To construct a lncRNA-miRNA-mRNA regulatory network, we combined existing miRNA and mRNA databases and used miRanda and Targetscan to predict the miRNA-binding sites of lncRNAs and mRNAs in the 45 vs. 75 comparison groups (Supplementary Table 2). 1,011 lncRNA-miRNA and 7,411 miRNA-mRNA interaction pairs were predicted, of which 449 lncRNAs and 316 mRNAs were involved in the construction of regulatory networks. Partial prediction results are shown in Figure 9.

### Dual-luciferase reporter assay

**Figure 10:**
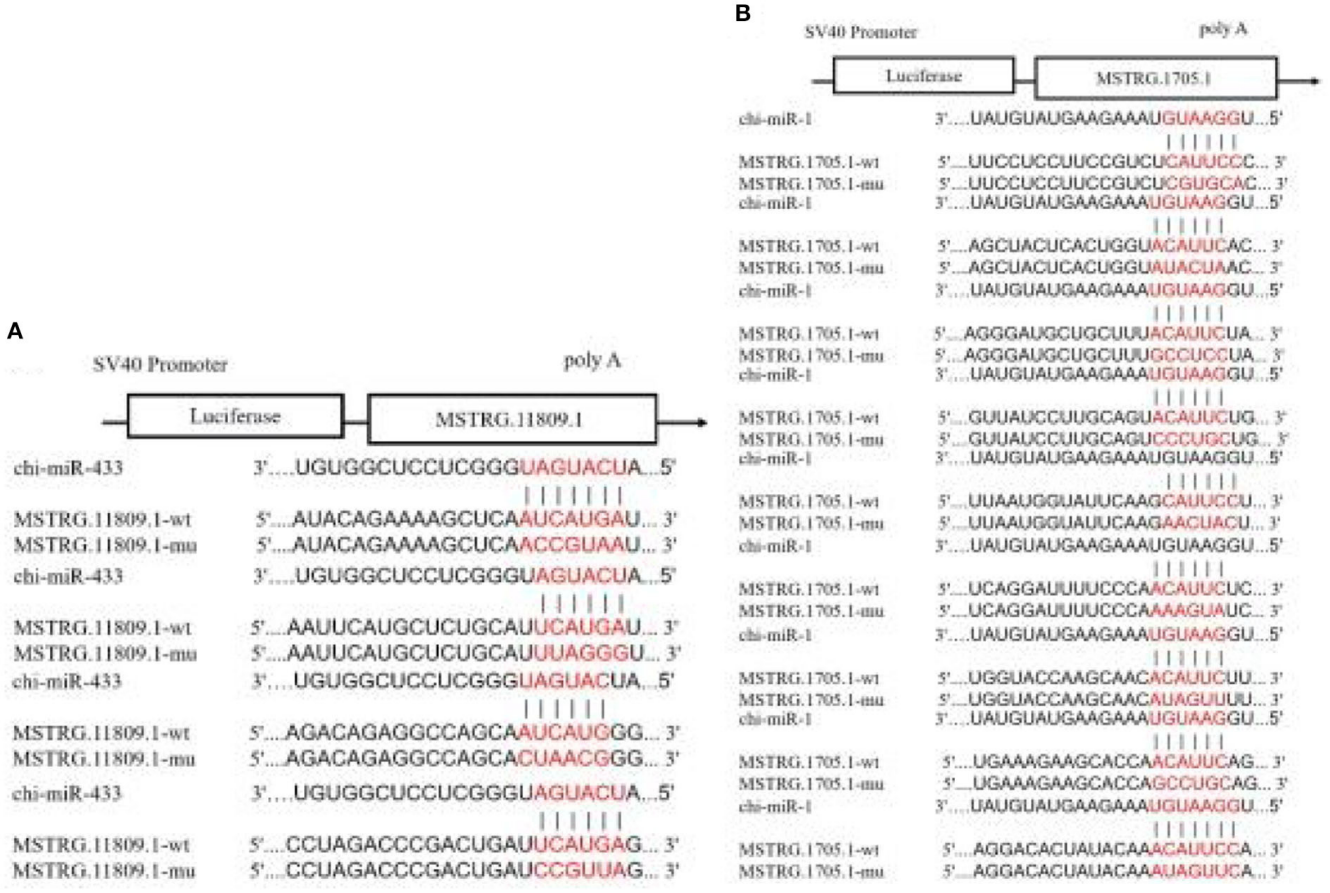
Prediction of lncRNA-miRNA binding sites and construction of vectors. **(A**) The predicted binding site and mutated site of *Chi-miR-1* in *lncRNA MSTRG.1705.1*. **(B)** The predicted binding site and mutated site of *Chi-miR-433* in *lncRNA MSTRG.11809.1*.

**Figure 11:**
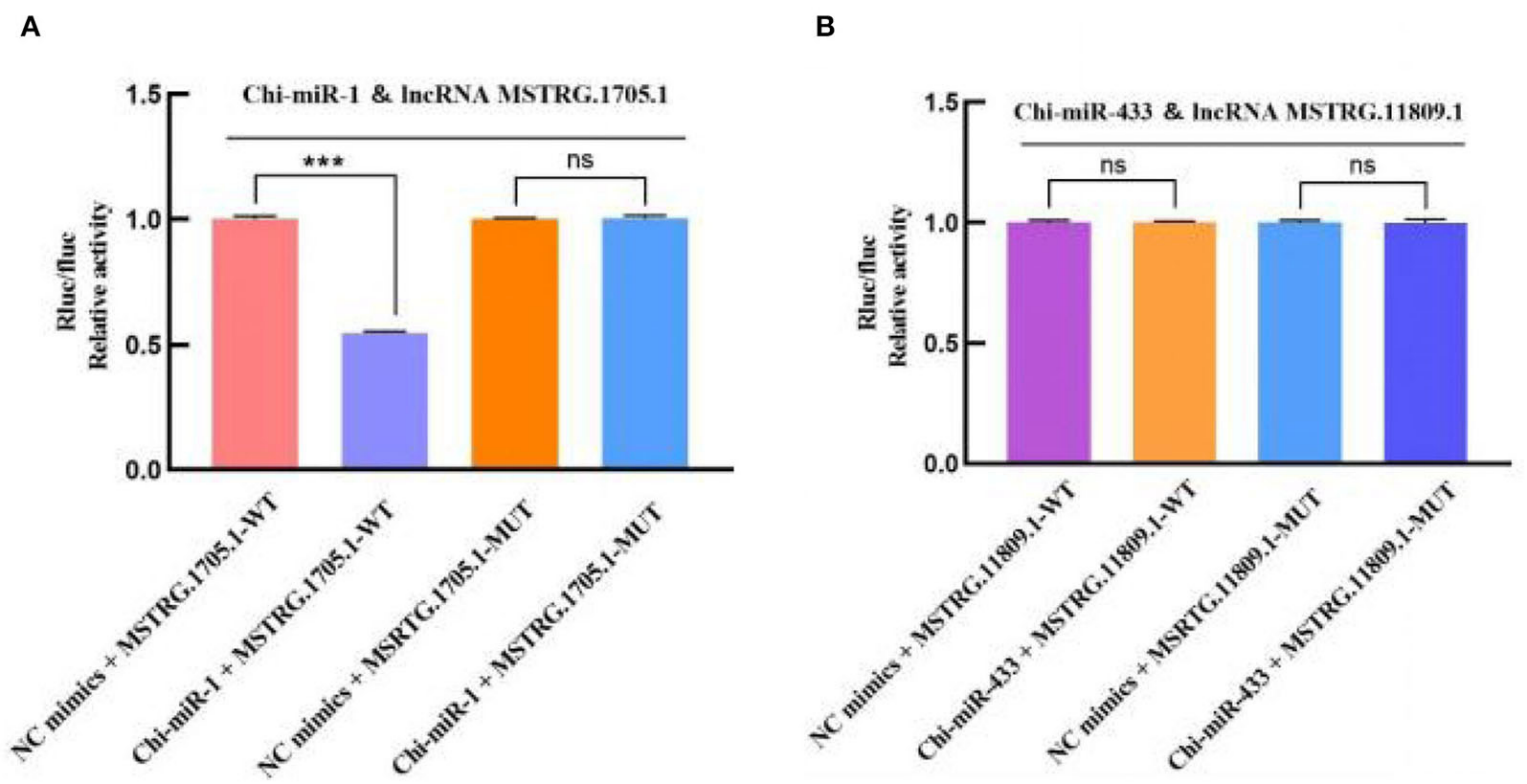
Dual-luciferase verification of the binding relationship between lncRNA-miRNA. **(A)** Detection of interaction between *lncRNA MSTRG.1705.1* and *Chi-miR-1* by dual-luciferase reporter gene assay. **(B)** Detection of interaction between *lncRNA MSTRG.11809.1* and *Chi-miR-433* by dual-luciferase reporter gene assay.

In the 45 vs. 75 comparison groups, *lncRNA MSTRG.11809.1* and *lncRNA MSTRG.1705.1* were significantly differentially expressed; and Targetscan and miRanda software predicted that *lncRNA MSTRG.11809.1* had a *Chi-miR-433* binding site, *lncRNA MSTRG.1705.1* had *Chi-miR-1* binding site. Therefore, psiCHECK2-lncRNA MSTRG.11809.1-WT/MUT and psiCHECK2-lncRNA MSTRG.1705.1-WT/MUT vectors were constructed to verify the binding relationship between lncRNA and miRNA (Figure 10). The dual luciferase results are shown in Figure 11, compared with the NC group, *Chi-miR-1* significantly reduced the expression of luciferase in *lncRNA MSTRG.1705.1*-WT; after mutating the binding site, *Chi-miR-1* did not significantly reduce the expression of luciferase in the *lncRNA MSTRG.1705.1*-MUT. However, in the *lncRNA MSTRG.11809.1*-*Chi-miR-433* experiment, after adding *Chi-miR-433* mimics, the expression of luciferase in both the *lncRNA MSTRG.11809.1*-WT and *lncRNA MSTRG.11809.1*-MUT groups was unchanged. The above experimental results indicated that *Chi-miR-1* could bind to *lncRNA MSTRG.1705.1*, while *Chi-miR-433* did not have a binding site for *lncRNA MSTRG.11809.1*.

### Gene enrichment analysis

**Figure 12:**
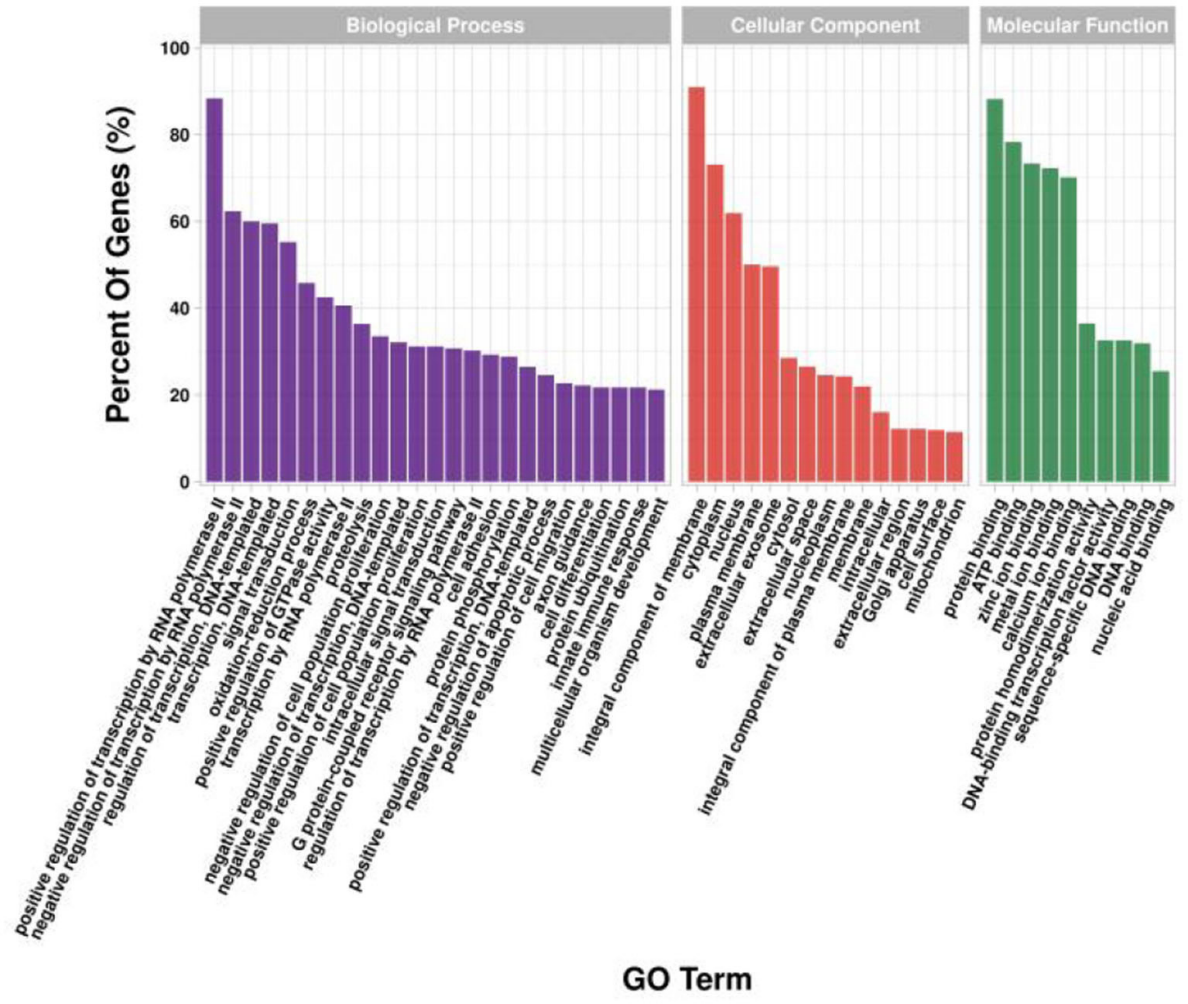
GO enrichment analysis of target genes in lncRNA regulatory network of hair follicle development in Inner Mongolia cashmere goats. The X-axis is the GO term, and the Y-axis is the enrichment significance.

**Figure 13:**
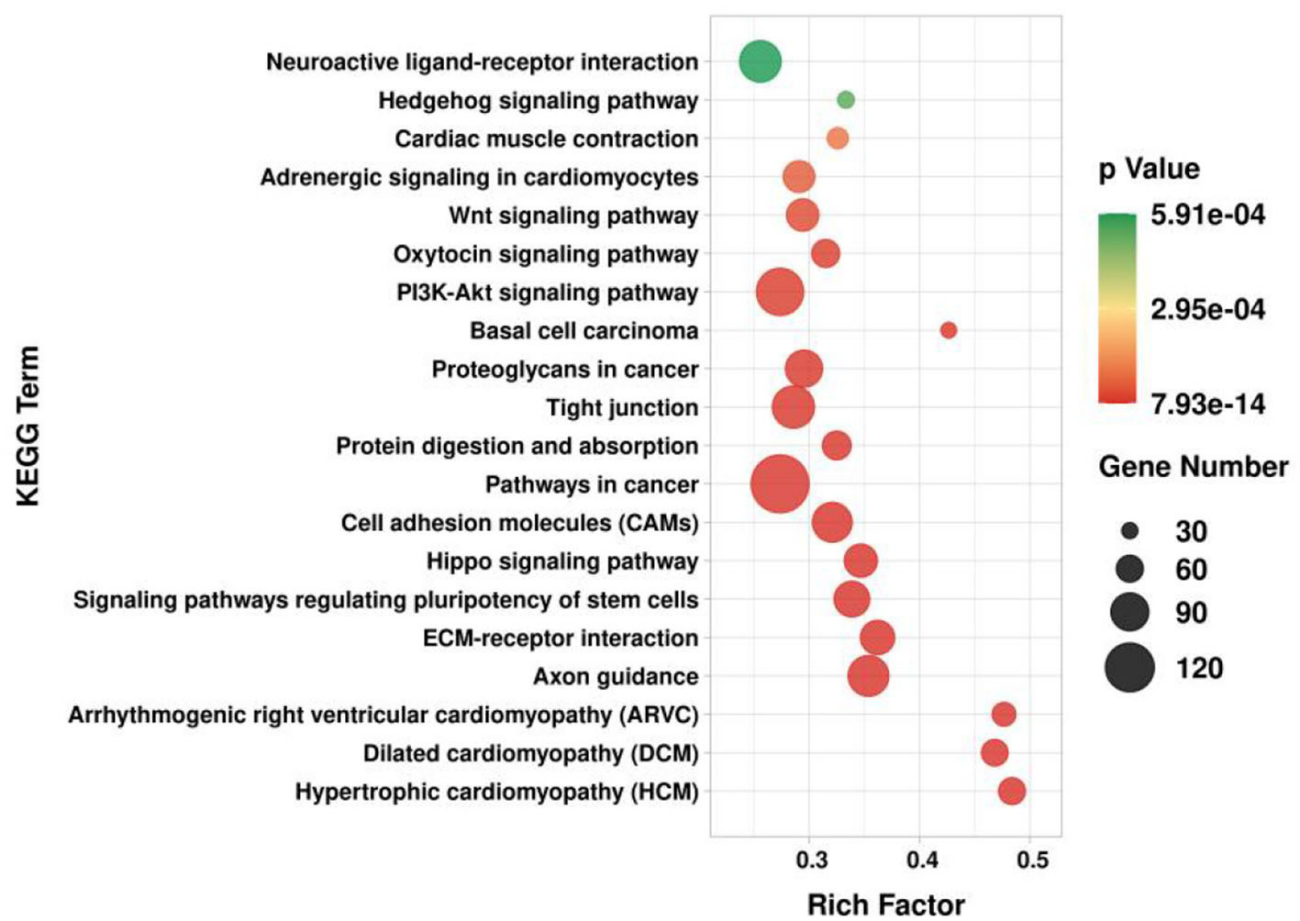
KEGG enrichment analysis of target genes in lncRNA regulatory network of hair follicle development in Inner Mongolia cashmere goats. The X-axis is the rich factor, and the Y-axis is the KEGG pathway.

GO and KEGG enrichment analysis was performed on target genes involved in lncRNA-mRNA and lncRNA-miRNA-mRNA molecular regulatory networks. The GO results showed (Figure 12) that in terms of biological processes, it was mainly enriched in positive regulation of transcription by RNA polymerase II (GO:0045944), positive/negative regulation of cell population proliferation (GO:0008284; GO:0008285), cell differentiation (GO:0030154), etc.; in terms of molecular function, it is mainly enriched in growth factor activity (GO:0008083), Wnt-protein binding (GO:0017147), β-catenin binding (GO:0008013), etc.; in terms of cellular component, it is mainly enriched in integral component of membrane (GO:0016021), cytoplasm (GO:0005737), cytoskeleton (GO:0005856), etc. The KEGG results showed (Figure 13) that target genes were enriched in the PI3K-Akt signaling pathway (ko04151) and Wnt signaling pathway (ko04310) related to hair follicle development. Therefore, the screened lncRNAs may directly or indirectly regulate genes related to hair follicle development, indirectly participate in cell proliferation, cell differentiation, and hair follicle development, and then play a regulatory role.

## Discussion

LncRNAs are widely involved in the life activities of animals, plants, and microorganisms, and their regulatory mechanisms are complex and diverse. Hair follicles are complex skin appendages that determine the yield and quality of cashmere (2). Their development in the fetus involves a series of interactions between the epidermis and dermis, and the process is relatively complex (3, 4). With the discovery of the function of lncRNA, the regulation of lncRNA on hair follicle development has also attracted the attention of researchers. In this study, the expression profiles of lncRNAs in hair follicles of Inner Mongolia cashmere goats at different embryonic stages (45, 55, 65, and 75 days) were constructed by high-throughput sequencing technology, and a total of 6,630 lncRNAs were identified. Through data analysis, it was found that most of the obtained lncRNAs were “i” and “u” types. In addition, by comparing the structural characteristics with the identified mRNA, it was found that the number and expression of lncRNA were far lower than the level of mRNA; lncRNAs also contain ORF and exon, but the number of them is much less than mRNA.

LncRNAs can interact with DNA, RNA, or protein at the transcriptional, epigenetic, and post-transcriptional levels to regulate gene expression, thereby regulating cellular activities such as cell cycle, differentiation, and metabolism (38). In this study, we combined the mRNA and miRNA database (published) to further explore the regulatory mechanism of lncRNA. The interaction between lncRNA and mRNA can be divided into two categories, one is cis-regulation, that is, lncRNA regulates the expression of its adjacent genes (within the upper and lower 100 kb range in the chromosome), and cis-regulation mainly depends on cis-acting elements, and then participate in the regulation of gene expression in the nucleus. the other type is trans-regulation, that is, lncRNA regulates the expression of genes across chromosomes, and trans-regulation mainly depends on the amount of free energy required for the formation of secondary structure between lncRNA and mRNA sequences. If only a very low free energy is required for the combination of the two sequences, there may be interactions between them. Due to the large results of lncRNA trans-regulation and the difficulty in later verification, only cis-regulation of mRNA and lncRNA was predicted in this study. We predicted a total of 516 lncRNA-mRNA cis-regulatory interaction pairs, of which 449 lncRNAs and 316 mRNAs were involved in the network. There are miRNA recognition element sites inside lncRNAs, which can act as ceRNAs to indirectly regulate gene expression. The ceRNA hypothesis was first proposed by Pier Paolo Pandolfi et al., that there are competing endogenous RNA (ceRNA) in cells, which can indirectly regulate mRNA expression by competing for binding sites of miRNAs (39). LncRNA as ceRNA involved in hair follicle development has become a research hotspot. Studies have found that *lncRNA-599547* can regulate the expression of *Wnt10b* gene by binding to *miR-15b-5p*, thereby inducing the differentiation of cashmere goat dermal papilla cells (40); *lncRNA 000679* can competitively bind to *miR-221-5p* with *Wnt3* and *lncRNA 000181* can competitively bind to *miR-34a* with *GATA3*, thereby indirectly regulating the growth of hair follicles (41). In this study, we predicted lncRNA-targeted miRNAs and miRNA-targeted mRNAs and obtained a total of 1,011 lncRNA-miRNA and 7,411 miRNA-mRNA interaction pairs, of which 401 lncRNAs, 24 miRNAs, and 3,611 mRNAs are involved in the network. In addition, we found that lncRNAs usually have binding sites with multiple miRNAs, and miRNAs also have binding relationships with multiple mRNAs, indicating that the regulatory network of ceRNA is complex and diverse, and the expression of a gene is often jointly regulated by multiple lncRNAs and miRNAs. Subsequently, *lncRNA MSTRG.11809.1* and *lncRNA MSTRG.1705.1*, which were significantly differentially expressed in hair follicles, were selected for dual luciferase assay. The results showed that *lncRNA MSTRG.1705.1* could bind to *Chi-miR-1*, while there was no binding site between *lncRNA MSTRG.11809.1* and *Chi-miR-433*.

Finally, we performed GO and KEGG enrichment analysis on the target genes involved in the regulatory network and found that they were mainly enriched in the Wnt signaling pathway and PI3K-Akt signaling pathway related to hair follicle development. Wnt is a cysteine-rich glycoprotein in the extracellular matrix, which transmits signals into the cell by interacting with frizzled receptors on the cell membrane, causing the accumulation of β*-catenin* in the cell and its transport into the nucleus. In the nucleus, β*-catenin* interacts with the Tcf/Lef family of transcription factors to achieve regulation of downstream genes. Previous studies have shown that the Wnt signaling pathway is widely involved in various aspects of hair follicle morphogenesis and plays an important role in hair follicle development (42). During mouse hair follicle development, *Wnt3, Wnt3a, Wnt4, Wnt6, Wnt7a, Wnt7b, Wnt10a*, and *Wnt10b* were all expressed in epithelial cells (14, 43). Transgenic mice overexpressing *Wnt3* exhibited phenotypes such as short hair, alopecia, and abnormal differentiation of hair follicles (44). β*-catenin* is a key protein in the Wnt signaling pathway. In mouse skin, overexpression of β*-catenin* induces the formation of new hair follicles; when the β*-catenin* gene is mutated, the normal pathway of hair follicle formation is blocked (45). The PI3K protein family is a series of kinases involved in cell growth, differentiation, proliferation, intracellular transport, and other processes, and its activation and inactivation play an important role in immunity, metabolism, and hair growth (46–48). Yamanami et al. (49) found that inhibition of the PI3K/Akt signaling pathway can reduce hair growth ability, while activation of the PI3K/Akt signaling pathway by an activator can increase the expression of genes related to hair growth, indicating that PI3K/Akt signaling pathway plays an important role in the maintenance and recovery of hair growth ability. Dermal sheath cells are considered to be the precursors of dermal papilla cells, and thrombin regulates the transformation between dermal sheath cells and dermal papilla cells through PI3K-Akt signaling pathways, thereby regulating the development of hair follicles (50). Melatonin is an important neuroendocrine regulatory factor, which can change the hair yield, color, and development of hair follicles in sheep, rabbits, rats, and other species (51, 52). Studies have shown that melatonin can promote the proliferation of dermal papilla cells and inhibit their apoptosis, which is achieved through the PI3K-Akt-mTOR pathway (53). According to the GO and KEGG enrichment results of target genes, we speculate that lncRNAs directly or indirectly regulate the genes related to hair follicle development, thereby regulating the proliferation, apoptosis, and differentiation of hair follicle cells. In the past few decades, there have been more studies on the regulation of miRNAs on hair follicles, while the reports on the regulation of lncRNAs on hair follicle development are relatively few (54–56). Therefore, this study explored the effects of lncRNAs on hair follicle development by constructing lncRNA expression profiles, which could provide the theoretical basis for further research on hair follicle morphogenesis and development, and provide important information for studying the mechanism of lncRNA in the human hair follicle.

## Conclusions

We constructed the expression profiling of lncRNAs in the hair follicles of Inner Mongolia cashmere goats at different embryonic stages (45, 55, 65, and 75 days), and predicted the molecular regulatory mechanism of lncRNAs by combining the screened miRNAs and mRNAs related to hair follicle development. Functional analysis showed that lncRNA mainly regulated hair follicle development through the PI3K-Akt signaling pathway and Wnt signaling pathway. The dual-luciferase results report that *lncRNA MSTRG.1705.1* was targeted to *Chi-miR-1*, while *lncRNA MSTRG.11809.1* was unbound to *Chi-miR-433*. In conclusion, this study provides an important basis for subsequent analysis of the molecular mechanism of lncRNA in the morphogenesis and development of the hair follicle of the cashmere goat.

## Data availability statement

The datasets presented in this study can be found in the SRA database (https://www.ncbi.nlm.nih.gov) under accession numbers: SRR13306938-SRR13306949.

## Ethics statement

The animal study was reviewed and approved by the Experimental Animal Ethics Committee of Inner Mongolia Agricultural University (Approval No. [2020] 056).

## Author contributions

RM, JL, and YZhan conceived the idea and designed the study. RM, FS, YR, JP, SN, YL, YQ, and MW participated in the sample collection. RM, FS, YR, JP, and MW performed the experiments. RM, QL, RW, and ZhiyW participated in the data analysis. RM wrote the draft. RM, FS, RS, ZL, YZhao, and XhixW finalized the manuscript. All authors read and approved the final manuscript.

## Funding

This study was supported by the Major Science and Technology Program of Inner Mongolia Autonomous Region (2021ZD0012) and the National Natural Science Foundation of China (31860627).

## Conflict of interest

The authors declare that the research was conducted in the absence of any commercial or financial relationships that could be construed as a potential conflict of interest.

## Publisher's note

All claims expressed in this article are solely those of the authors and do not necessarily represent those of their affiliated organizations, or those of the publisher, the editors and the reviewers. Any product that may be evaluated in this article, or claim that may be made by its manufacturer, is not guaranteed or endorsed by the publisher.

## Supplementary material

Supplementary Table 1 lncRNA-mRNA regulatory network.

Supplementary Table 2 lncRNA-miRNA-mRNA regulatory network.
